# Integrating Ecofeminism Into Canadian Nursing to Tackle Climate Change and Health Issues

**DOI:** 10.1111/nin.70035

**Published:** 2025-05-26

**Authors:** Émilie Tremblay, Sandra Harrisson

**Affiliations:** ^1^ School of Nursing University of Ottawa Ottawa Ontario Canada

**Keywords:** climate change, collective action, ecofeminism, environment, human health, nursing, nursing metaparadigm, psychoterratic syndromes

## Abstract

This paper presents an overview of the health impacts associated with anthropogenic climate change and examines the interconnection between human health and the environment. It highlights the nursing profession's stance on environmental issues, drawing attention to the disengagement of nurses from advocacy initiatives related to climate change and how this relates to the nursing metaparadigm. Moreover, this paper supports a multidirectional approach to address climate change solutions, with a particular emphasis on both adaptation and mitigation strategies. Ecofeminism is proposed as a critical framework to address the shortcomings of the metaparadigm and the approaches to climate change solutions. It examines the potential for integrating ecofeminism into nursing research and practice by reconceptualizing the concept of the environment, adopting an ethic *of* the environment, and critiquing oppressive social structures. The benefits of ecofeminism for nursing include enhancing nurses' responsiveness to the health consequences of climate change, facilitated by using a critical voice that promotes inclusion and collective action.

## 1 Background

**Figure 1:**
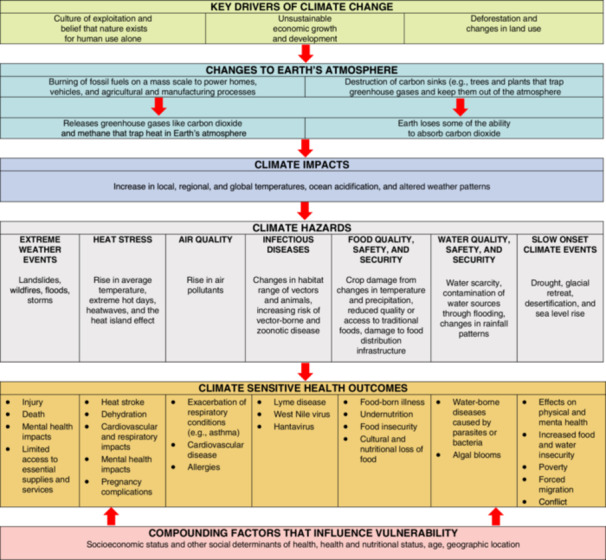
Impacts of Climate Change on Health. Source: ©All rights reserved. CPHO's Report on the State of Public Health in Canada, 2022: Mobilizing Public Health on Climate Change in Canada. Public Health Agency of Canada, modified: 2024‐02‐19. Adapted and reproduced with permission from the Minister of Health, 2025. Available at: Full report: Mobilizing Public Health Action on Climate Change in Canada: Chief Public Health Officer's Report on the State of Public Health in Canada 2022 – Canada. ca.

Anthropogenic climate change refers to the rapid warming of the climate due to excessive emissions of greenhouse gases (GHGs) into the atmosphere, along with environmental degradation resulting from unsustainable human resource production and consumption (Intergovernmental Panel on Climate Change 2023). Climate change‐related health impacts highlight the relationship between the environment and humans (Intergovernmental Panel on Climate Change 2023; Lawrance et al. 2022; Romanello et al. 2024) (see Figure 1). For example, wildfires arising from warmer temperatures and drier conditions exacerbate diseases of the lungs, heart, nervous system, skin, liver, kidneys, eyes, and nose due to smoke, which produces high levels of air pollution (Perrotta 2019; World Health Organization [WHO] 2024a). Additionally, awareness of climate impacts and personal encounters with climate‐related disasters have been linked to stress, anxiety, depression, and posttraumatic stress disorder (Charlson et al. 2021; Clayton et al. 2021; Hickman et al. 2021; Lawrance et al. 2022). Furthermore, climate change acts as a threat amplifier, exacerbating existing societal inequities, such as racism, poverty, disability, colonialism, homelessness, gender, and access to healthcare (Health Canada 2022; Hwong et al. 2022; Lawrance et al. 2022; Romanello et al. 2024). Vulnerable and marginalized groups, including children, seniors, and racialized communities, are at a higher risk of facing adverse health consequences due to climate change.

Climate change research has focused on health outcomes and less on adaptation and mitigation (Harper et al. 2021). While understanding health outcomes is vital to addressing climate change, proposed solutions require implementing both adaptation and mitigation strategies (Intergovernmental Panel on Climate Change 2023; Romanello et al. 2024). Therefore, a multidirectional approach is needed. Nurses' educational background and social mandate to promote health make them key change agents in this effort (Canadian Association of Nurses for the Environment & Canadian Nurses Association 2024). Some Canadian nurses are already implementing adaptation strategies at the individual level by educating their patients about illness prevention and the resources available to protect themselves against climate change (Canadian Federation of Nurses Union, & Canadian Association of Nurses for the Environment 2023). Still, they lack engagement at the higher sociopolitical level (e.g., governments and private sector activities) to address mitigation strategies.

We suggest that nurses adopt ecofeminism as a framework to address the sources of environmental pollution that have exacerbated health problems. By applying the proposed critical ecofeminist analysis, nurses will have compelling arguments to advocate for individuals whose physical and mental well‐being is adversely affected by climate change, especially those who cannot speak, articulate their needs, or have limited options.

## 2 Nursing and Environmental Issues

In 1984, Fawcett declared the four central concepts of the nursing discipline's metaparadigm: person, environment,^1^ health, and nursing care. The intent was to orient nursing's research agenda to reflect the profession's core values, guide nurses' systematic thinking about nursing and its practice, and communicate their professional beliefs, assumptions and caring actions (Fawcett 1984; Thorne et al. 1998). Of these four concepts, three recurring themes in the literature identify phenomena relevant to the discipline (Fawcett 1984). One of these is the relationship between person, environment, and health. Environmental issues, such as climate change, are indeed phenomena of research interest to the nursing discipline as they inform the practice of caring for affected patients, reflecting the profession's intention.

Similarly, many Canadian nursing associations believe that nurses' roles regarding environmental health issues, like climate change, include educating the public on these matters, reducing environmental harm, collaborating with colleagues to identify and mitigate environmental health risks, advocating for policies that protect health by preventing exposure to hazards, and promoting sustainability (Canadian Association of Nurses for the Environment & Canadian Nurses Association 2024; Canadian Association of Nurses for the Environment 2024; Canadian Nurses Association 2017; Registered Nurses’ Association of Ontario 2024). Nonetheless, there appears to be a gap between the expectations set by professional nursing associations and reality. For instance, Kalogirou, Dahlke, et al. (2020) found that Canadian nurses' knowledge of climate change varied, with their understanding of the causative factors and outcomes ranging from limited to well‐informed. While most nurses in the study viewed climate change as a personal concern, their role in addressing the issue as nurses was not clearly understood (Kalogirou, Dahlke, et al. 2020).

Current and future nurses should receive proper education on the causes and effects of climate change to prepare them for practice. However, few resources are available to guide curriculum development and support knowledge acquisition (Neal‐Boylan et al. 2019; Portela Dos Santos et al. 2023; Stephens and Leslie 2024), presenting a challenge globally and within Canada. As a result, nurses lack “eco‐literacy,” which pertains to their awareness of environmental issues and how to prevent them (Kalogirou, Olson et al. 2020; Portela Dos Santos et al. 2023; Stephens and Leslie 2024). Limited eco‐literacy has contributed to the profession's disengagement, feelings of demotivation or overwhelm, and uncertainty about how to act (Portela Dos Santos et al. 2023). This is worrisome because, as the Canadian Nurses Association stated in 2017, the public expects nurses to understand how to promote and support actions that optimize human health in the context of climate change.

Nurses' eco‐literacy can be traced back to the nursing metaparadigm, which has faced criticism for its narrow conceptualization of the environment that no longer corresponds to the current context (Kalogirou, Olson, et al. 2020; Portela Dos Santos et al. 2023; Thorne et al. 1998). Consequently, nurses cannot tackle significant societal environmental health issues like climate change. Ecofeminism has the potential to broaden nurses' perception of the relationship between humans and the environment and provide a basis to critique institutions, norms, and ways of thinking to alleviate health inequities and unequal societal privileges.

## 3 Ecofeminism

Ecofeminism, first identified in 1974 by feminist thinker Françoise d'Eaubonne, emerged from a deconstruction of grand narratives of oppression rooted in market economies combined with ecological theory (Salleh 1996; Warren 1990). One main principle of ecofeminism is to preserve and protect nature, which contradicts conventional economic measures that overlook the efficient use of natural resources. Ecofeminism highlights essential links between women, men, nature, and culture within patriarchal societies, with varying emphases and frameworks (Mies and Shiva 1993; Plumwood 1993; Salleh 1996; Warren 1990, 2000). For instance, some theorists focus on capitalism and colonialism (Mies and Shiva 1993; Salleh 1996), social justice (Salleh 1996), the critique of dualisms between men and women (Plumwood 1993), or the logic of domination (Plumwood 1993; Warren 1990, 2000).

Ecofeminism is an action‐oriented philosophy that seeks to deconstruct discriminatory and exploitative social practices. The central ideas of ecofeminism summarized by Besthorn and McMillen (2018) are as follows: (1) The dynamics of unequal power and exploitation between masculinity and femininity reflect the division between humans and nature; (2) Oppressive conceptual frameworks that maintain relations of domination are feminist concerns; therefore, ecological critiques are incomplete without an assessment of unchecked power and its effects on society; and (3) Humanity has lost awareness of human and nonhuman interdependence due to the institutions of modernity.

### 3.1 Warren's Ecofeminism

Warren's perspective aligns with the core concepts of ecofeminism. For instance, it connects the oppression and domination of women and nature to address environmental issues such as climate change while advocating for the moral consideration of nature by humans (an ethic *of* the environment) and highlighting the interconnectedness of all living beings. Nevertheless, her perspective sets itself apart from other ecofeminists because, rather than focusing solely on women and nature, she recognizes all those unjustifiably dominated or subordinated without implying any biologically determined or socially constructed reasons. Warren (2000) argues that her ecofeminist perspective is “feminist” because feminism clarifies the connection between the domination of nature and the subordination and oppression of women and other humans to patriarchy.

Warren (2000) ecofeminism draws inspiration from feminism, ecology, environmentalism, and philosophy. It begins with a sex/gender analysis to criticize human systems that enable unjustified domination, known as “isms of domination” (Warren 2000), including sexism, naturism, ageism, classism, racism, heterosexism, ableism, anti‐Semitism, and colonialism. Next, ecological and environmental knowledge and the human‐nature interaction are employed in both theory and practice (Warren 2000). As a philosophy, ecofeminism uses conceptual analysis to examine and understand the historical functions of different systems of oppression (e.g., conceptual frameworks). It uses argumentative justification to question the rationality of discourses of domination, recognizing that oppressive conceptual links do not sanction subordination (Warren 2000).

A conceptual framework consists of beliefs, values, attitudes, and assumptions through which a person views themselves and the world (Warren 2000). It “functions as a socially constructed *lens* through which one perceives reality.” (Warren 2000, 46). Conceptual frameworks are shaped and influenced by various factors, such as sex/gender, age, race/ethnicity, class, orientation, marital status, religion, nationality, colonial influences, and culture (Warren 2000). It is deemed oppressive when it explains and justifies relationships of unjustified domination and subordination (Warren 1990, 2000).

An oppressive conceptual framework has five fundamental characteristics (see Table 1) and is considered patriarchal if it justifies the subordination of women by men based on gender (Warren 2000). This occurs through institutions (policies, practices, positions, roles), norms, and ways of thinking (conceptual frameworks), which accord men higher value, status, prestige, and power than women. The logic of domination is a central aspect of an oppressive conceptual framework, as it unjustly legitimizes superior status, power and privilege to men over women, thereby maintaining unjust relationships of domination and subordination (Warren 2000). The same foundations legitimize domination and subordination among individuals and between humans and nonhuman nature (Warren 2000). In other words, the oppression of women, people of color, children, and people with low incomes within patriarchy is linked by a common logic of domination.

**Table 1:** Five characteristics of an oppressive conceptual framework (Warren 2000, 46‐47).

Value‐hierarchical “Up‐Down” thinking	The value‐hierarchical thinking assigns greater value to what is superior, or Up, than to what is inferior, or Down. By attributing greater value to what is superior, the Up‐Down organization of reality serves to legitimize inequality.
Opposing value dualisms	Oppositional value dualisms are disjunctive pairs that are considered exclusive and oppositional, rather than inclusive and complementary, while a greater value (status and prestige) is accorded to one disjunction than to another.
Power	The power that is conceived and exercised as “power over”. In an oppressive system, “power over” serves to reinforce the power of Ups as Ups in a way that keeps the Downs in unjustified subordination.
Privilege	The practice of privilege is that which belongs to Ups and not Downs. When the privilege of Ups serves to maintain intact the dominant‐subordinate relations between Ups and Downs that systematically advantage Ups over Downs, it is part of a set of oppressive practices.
Logic of domination	The logic of domination is an argumentation structure that justifies the domination of the Down by the Up. Such a logic offers moral approval for subordination and assumes that superiority justifies subordination.

The term “Others” is often used to describe individuals who deviate from the ideal dominant societal norm (Avanthay Strus et al. 2024). This perspective considers the White, able‐bodied, bourgeois, heterosexual, and masculine body as the standard, embodying a higher status and representing the pinnacle of control and civility. That said, Warren (2000) uses “Others” to highlight the status of subordinate groups within unjustifiable relationships and systems of domination and subordination, which encompass both “human Others” (e.g., women, people of color, children, and people with low incomes) and “earth Others” (e.g., animals, plants, ecosystems, forests, and land). Thus, Warren's ecofeminism identifies equity‐denied and marginalized groups as “Others”. This includes gender minorities (e.g., those who identify as gender nonbinary), nonheterosexual individuals, itinerant or homeless individuals, non‐whites, Indigenous peoples, and people with disabilities.

### 3.2 Ecofeminism and Environmental Issues

Warren's conceptual analysis and argumentative justification allow us to grasp how oppressive conceptual frameworks function within society. We can start to challenge hierarchical “Up‐Down” thinking, which legitimizes the depletion of natural resources for economic gain, as cultural and societal institutions are perceived as Up while nature and the environment are seen as Down (Warren 1990, 2000). This understanding highlights that in a patriarchal society, nature is valued primarily for the resources it provides for the material production of goods, which is included in the national economic gross domestic product (GDP) (Warren 1990, 2000). Such insights equip us with the means to deconstruct ideologies and critique the numerous consequences that oppressive patriarchal structures impose on the natural environment, nonhuman nature, and humans, while also motivating us to take personal and political action against them.

For instance, Warren's ecofeminism can serve as a basis for critiquing commercial determinants of health. The World Health Organization defines commercial determinants of health as “a key social determinant, referring to the conditions, actions, and omissions by commercial actors that affect health” (2023). These commercial activities adversely impact physical and social environments, contributing to issues like intensive animal agriculture, deforestation, and pollution of air, soil, and water, along with commercial efforts in knowledge sectors that fuel climate change denialism (WHO 2023). Additionally, these companies often sway public health through lobbying and party donations (WHO 2023), efforts that have aimed to weaken environmental regulations that affect their products and services. Although it is widely known that corporate influence in political spheres hinders climate action (Lawrance et al. 2022), an ecofeminist critique helps to dissect the complex issue and provides us with justified arguments to advocate for health and social justice.

## 4 Nursing and Ecofeminism

Ecofeminism can assist nurses in incorporating climate strategies into their practice by broadening their perspective of the environment, adopting an ecofeminist ethic *of* the environment, and providing a framework to critique institutions, norms, and ways of thinking to alleviate health inequities and unequal societal privileges.

### 4.1 Reconceptualizing the Nursing Metaparadigm's Environment Concept

While the metaparadigm is not overtly oppressive, it limits nurses' ability to address environmental health issues such as climate change. The concern lies in its narrow interpretation of the environment as rigid, static, and natural, instead of fluid, changeable, and constructed (Kalogirou, Olson, et al. 2020; Thorne et al. 1998).

First, the nursing metaparadigm focuses solely on the individual, not on society (Kalogirou, Olson, et al. 2020; Kleffel 2006; Portela Dos Santos et al. 2023; Thorne et al. 1998). In this sense, the metaparadigm has led nurses to consider only the factors that affect the human‐environment relationship within their immediate surroundings. This is problematic since individual experiences can be isolated from social, ecological, economic, or political realities (Thorne et al. 1998), and if the environment is solely defined as one person's perception, slow‐progressing environmental issues like climate change are almost impossible to grasp (Kalogirou, Olson, et al. 2020). Climate change affects everyone, though not equally, and will exacerbate health, social, and economic inequities (Health Canada 2022; Lawrance et al. 2022; Romanello et al. 2024). Therefore, social determinants of health—such as structural barriers like racism, pre‐existing health conditions, socioeconomic status, education, and access to care—must be considered since some groups are more vulnerable than others to the impacts of climate change. Furthermore, theories that address complex problems like ecological health highlight that individual actions are insufficient (Huttunen and Albrecht 2021; Kalogirou, Olson, et al. 2020). There is a need for collective action and a focus on politics because it is at the higher societal level where these issues originate (i.e., the root cause of climate change).

Second, in the nursing metaparadigm, individuals cannot change their environment and must adapt to it (Kalogirou, Olson, et al. 2020; Portela Dos Santos et al. 2023; Thorne et al. 1998). In this sense, the metaparadigm suggests that all environmental conditions are considered “natural” (i.e., not caused by human systems). This is erroneous since anthropogenic climate change arises from human activities that impact the environment (Intergovernmental Panel on Climate Change 2023). While an essential approach to climate change includes adaptation strategies, such as educating the public about preventing Lyme disease from tick bites (Canadian Federation of Nurses Union, & Canadian Association of Nurses for the Environment 2023), it is limited when discussed without mitigation. Mitigation strategies aim to minimize environmental harm caused by humans, such as reducing GHG emissions (Romanello et al. 2024), which exacerbate health problems in the first place. Without these strategies, health inequalities and inequities will worsen due to their harmful effects on socioeconomic determinants of health. Nurses must move beyond concentrating only on individual behaviors, as this limits their ability to comprehend and influence societal mindsets and the policies that permit harmful environmental practices contributing to climate change.

Warren's ecofeminist analysis helps nurses reconceptualize the environment as fluid, changeable, and constructed by examining how institutions, norms, and ways of thinking shape reality. Ecofeminism highlights how human institutions have justified harmful environmental practices at the expense of environmental and human health. This perspective allows nurses to view the environment as something they can influence or as something their healthcare practices can affect. It advances a sociopolitical outlook and emphasizes the importance of mitigating climate change. Moreover, ecofeminism affirms that one's existence is shaped and influenced by various factors such as sex/gender, age, race/ethnicity, class, orientation, marital status, religion, nationality, colonial influences, and culture (Warren 2000). Nurses can provide sensitized care by considering multiple realities and meaningfully examining social, economic, and political variables that affect their patients' lives. This is particularly important because people and communities are more susceptible to climate change due to income, culture, and education (Health Canada 2022). Population‐based knowledge will allow nurses to understand how people are connected to their environment and how it impacts their daily lives, health, and well‐being.

### 4.2 Ethic of the Environment

Warren's ecofeminism emphasizes that humans should treat nature according to an “ethic *of* the environment” which stipulates that the natural nonhuman environment deserves moral consideration (2000). Thus, nature and all life forms are intrinsically valuable due to their inherent properties, not because of their usefulness for some purpose or end. Moral consideration is based on being alive, as humans and other species are multiple beings that coexist and work towards a common interest: life. An ethic of the environment aligns with an ecocentric view that considers nature not simply as a resource, property, or commodity, but as something deserving of our care and being valued even if humans cannot use it as a resource (Kleffel 2006; Terry et al. 2019; Warren 2000). By adopting an ethic of the environment and an ecocentric view (as opposed to an egocentric view), nurses promote collective action over individuality. They encourage humans to govern themselves with respect for all reciprocal relationships in a sociobiological system at all scales (Albrecht 2019, 2020). It is not only morally sound for nurses to do so but necessary because the health and wellness of communities and the environment depend on humanity moving towards a new era characterized by interconnectedness.

### 4.3 Critiquing Oppressive Social Structures

Critiquing oppressive social structures that justify the unequal distribution of opportunities and privileges lays the groundwork for nurses to suggest alternative, collective, and inclusive ways of knowing and doing to address environmental health issues. To achieve this, the functioning of individualism, an adult‐centered viewpoint, and rationalism is examined through an ecofeminist perspective.

#### 4.3.1 Individualism Versus Collectivism

Ecofeminist philosophy assumes that people are relational selves, socially embedded with one another and the environment (Warren 2000). Therefore, it rejects the view of “abstract individualism,” which aligns with an egocentric view (Kleffel 2006), where people are essentially solitary, separate, and isolated individuals, defined apart from their social contexts and relationships (Warren 2000). Warren's conceptual analysis contributes to understanding how neoliberal policy agendas promote dependence on economic growth, regardless of environmental or health implications, while stressing individual responsibility for personal well‐being and ecological health (Chircop 2008). When emphasis is placed on individualism, responsibility shifts away from societal structural factors that significantly influence the climate crisis (Chircop 2008). This situation is particularly alarming for people living in poverty, most of whom are women, especially single mothers and their children (Government of Canada 2024; Statistics Canada 2024). Individuals with already vulnerable health due to their income must navigate health challenges with limited resources. Compounding this difficulty are the harmful practices they did not contribute to, which is disheartening. Nevertheless, individual actions are insufficient to address climate change and health‐related climate impacts (Chircop 2008; Galway and Field 2023; Huttunen and Albrecht 2021; Stern et al. 1999).

Recognizing that sociopolitical and economic contexts and underlying ideologies influence everyday realities (Health Canada 2022; Romanello et al. 2024; Warren 2000), a critical ecofeminist lens helps nurses uncover oppressive social structures constraining people's health and restricting their equal and conscious participation in society. Nurses can challenge these structures by promoting collective action instead of individualism and advocating for environmentally sustainable policies. By taking these steps, nurses can alleviate the unjust health burden placed on individuals and shift responsibility toward the causes of adverse health reactions (i.e., fossil fuel air pollution).

#### 4.3.2 Adults Versus Youth and Future Generations

The effects of climate change will impact younger generations (and future generations) more than older ones (Chamila Roshani Perera and Rathnasiri Hewege 2013; Cunsolo and Ellis 2018; Huttunen and Albrecht 2021). Many young people experience grief and strong emotions from growing up with ‘doom and gloom’ narratives about an uncertain future (Cunsolo and Ellis 2018), including substantial risks to health, food security, housing, and natural ecosystems (Huttunen and Albrecht 2021; Perrotta 2019). While future generations – those yet to be born – do not have a say in the matter, the voices of young people are often discouraged or ignored in institutional forms of environmental political participation, such as elections and political parties (Chukwudozie et al. 2015; Galway and Field 2023; Hickman et al. 2021; Shamrova and Cummings 2017). This is ironic since older generations have the power and privilege to make decisions with severe ecological consequences. Still, the younger generation is left to deal with the consequences imposed on them by these decisions.

Ecofeminism's value hierarchical Up‐Down thinking (Warren 2000) reveals how the older generation justifies large‐scale exploitation and depletion of natural resources, as well as the exclusion of young people from political participation. This justification takes the form of a characteristic that the Up has and that the Down lacks (Warren 2000). Simply put, adults (Ups) have greater intellectual maturity than children (Downs), and therefore, ageist assumptions are maintained to ignore young people when they raise valid concerns about climate change and its threat to human health and the environment. Ecofeminism empowers nurses to challenge misconceptions about young people's unjustified inferiority to adults, advocate for their right to be heard by the older generation in power, and to be involved in proposing and implementing climate change strategies. Young people have a longer life expectancy; they deserve a healthy environment and the right to self‐determination.

#### 4.3.3 Rational Versus Emotional

Warren (2000) ecofeminism rejects the traditional objective point of view, suggesting that there is only one value‐neutral, detached, or impartial way of knowing. Her ecofeminism reveals the historical functions and associations of opposing value dualisms and their long‐lasting effects that continue to impact the present context. Historically, higher status has been accorded to “male,” “white,” “rational,” and “culture” than to what has traditionally been identified as “female,” “black,” “emotional,” and “nature” (Warren 2000). According to these beliefs, it is better to be rational than emotional. Additionally, being emotional is associated with women and nature, while being rational is associated with men and culture.

Mental health refers to an individual's emotional well‐being and psychological state (Public Health Agency of Canada 2020). Interestingly, research has traditionally favored objective evidence (“rational” or “factual”) over subjective evidence (“emotional” or “psychological”) (Coffey et al. 2021; Harper et al. 2021; Holmes et al. 2006). This is reflected in published studies on physical and mental climate‐related health impacts. While physical health impacts are thoroughly researched (Harper et al. 2021; Intergovernmental Panel on Climate Change 2021, 2023), fewer studies focus on mental health (Harper et al. 2021; Hwong et al. 2022; Lawrance et al. 2022). This is concerning, as the effects of climate change are expected to continue increasing (Intergovernmental Panel on Climate Change 2023), along with the distress related to awareness of its threat (Lawrance et al. 2022).

Nursing stands to benefit from an ecofeminist perspective as it promotes inclusivity and rejects the idea that there is only one way of knowing. This framework does not prioritize physical over mental health, or vice versa, aligning with the World Health Organization's comprehensive view of health that encompasses physical, mental, and social well‐being (2024b). By promoting research and education to understand the impacts of climate change on both physical and mental health, and how they interrelate, nurses can validate people's experiences, provide support, and adequately respond to their overall health needs instead of focusing on one aspect while overlooking others. Integrating ecofeminism into nursing will help nurses care for individuals whose well‐being is connected to the natural environment and is affected by slow, progressive, and large‐scale environmental changes.

### 4.4 Psychoterratic Syndromes

The term “psychoterratic syndromes” (or “climate emotions”) refers to psychological and emotional responses where a person's mental well‐being is affected by the environmental changes to which they are exposed, directly or indirectly (Albrecht 2019; Albrecht et al. 2007; Cunsolo and Ellis 2018; Galway et al. 2019; Pihkala 2020). Responses like ecological grief, eco‐anxiety, and solastalgia describe how someone feels despair, anxiety, and distress when confronted with the experienced or anticipated loss of species, the perceived threat of large‐scale climate disasters, or the destruction of their homeland (Albrecht 2006; Cunsolo and Ellis 2018; Pihkala 2020), whether due to past natural disasters, current ecological losses, or uncertainty about the future.

Health professionals and researchers have avoided pathologizing these psychological and emotional reactions because, for most people, psychoterratic syndromes are rational and appropriate responses to the threat of the climate crisis, representing a healthy and adaptive process (Comtesse et al. 2021; Cunsolo and Ellis 2018; Hickman et al. 2021; Lawrance et al. 2022; Pihkala 2020; Soutar and Wand 2022). Emerging research has found that engaging in personal and collective climate actions and fostering strong community networks are valuable coping mechanisms for challenging climate emotions, such as worry and anxiety (Galway and Field 2023; Lawrance et al. 2022). However, some individuals have maladaptive responses, resulting in their climate emotions significantly impacting their daily functioning (Comtesse et al. 2021; Galway and Field 2023; Lawrance et al. 2022), making it challenging to cope with these effects independently. In these situations, professional psychological support should be considered.

Mental health research is advancing to understand risks associated with immediate direct impacts, such as floods or heatwaves, and longer‐term direct impacts, such as forced migration and loss of livelihoods (Charlson et al. 2021; Hwong et al. 2022; Massazza et al. 2022; Soutar and Wand 2022), resulting in psychoterratic syndromes. However, there remains a lack of data on the prevalence, nature, and severity of psychological and emotional experiences stemming from indirect climate change impacts (Cunsolo and Ellis 2018; Hwong et al. 2022; Lawrance et al. 2022) that lead to psychoterratic syndromes—for instance, witnessing changes to landscapes and loss of ecosystems (Albrecht et al. 2007; Cunsolo et al. 2020; Cunsolo and Ellis 2018), awareness of slow, gradual environmental degradation, and future uncertainty (Clark 2020; Clayton et al. 2021; Coffey et al. 2021; Pihkala 2020). As a result, the public (Cunsolo and Ellis 2018) and political leaders (Cunsolo and Ellis 2018; Lawrance et al. 2022) often fail to acknowledge the rational adaptive responses to the indirect impacts of climate change as a mental health concern. Thus, the extent of mental health impacts from climate change is neither fully understood nor adequately addressed in health and environmental policies. We suggest using ecofeminism to reveal the factors influencing the physical and mental health of children, adults, older populations, and community health in the context of climate change.

## 5 Conclusion

Shifting accountability for health and well‐being from individuals to structural determinants is imperative. We must use an inclusive and collective voice that accounts for factors such as sex, gender, age, race, ethnicity, socioeconomic status, sexual orientation, marital status, religion, nationality, colonial influences, and culture. Despite the nursing profession's shortcomings, Warren's ecofeminist framework can be integrated into nursing, facilitating an understanding of the connections between environmental and human health, thus framing climate change as a societal concern rather than an individual predicament. On the one hand, ecofeminism underscores the importance of rebuilding our interdependent relationship with the environment. This transition involves shifting from an egocentric perspective to an ecocentric one, emphasizing interconnectedness. Conversely, it provides nurses with a legitimate and critical voice to challenge the oppressive processes of patriarchal systems that significantly influence social, political, economic, ecological, and health outcomes. It's about time that we, as a society, stop allowing the financial success of a minority of wealthy individuals who have the resources to protect themselves against climate change and start focusing on actions that favor the health of the majority. This directly contrasts with Big Oil's bottom line by preventing the release of hazardous materials into the environment that make people sick and cause environmental degradation.

## Conflicts of Interest

The authors declare no conflicts of interest.

## Data Availability

The authors have nothing to report.

## Endnotes

1 In this paper, the environment refers to nature, landscapes, and ecosystems around us. The human–environment relationship is bidirectional: human sociopolitical and economic structures affect the environment, and humans are influenced by the environmental conditions they experience and live in.
